# Prevalence and Factors Associated with Maternal Postpartum Depression among Mothers in Saudi Arabia: A Cross-Sectional Study

**DOI:** 10.3390/healthcare11030343

**Published:** 2023-01-25

**Authors:** Amani Osman Abdelmola, Ahmed Bahari, Ibrahim Gosadi, Khdeeja Shami Aburasain, Njoud Ali Osaisi, Nidaa Sameer Jilan, Sarah Rashad Alsanosy, Haneen Ali Mahnashi, Hadeel Fuad Gadri, Amnah Ahmad Khobrani, Alaa Ahmad Darraj, Mohamed Salih Mahfouz, Hadi Dhafer Hassan Kariri, Siddig Ibrahim Abdelwahab

**Affiliations:** 1 Department of Family and Community Medicine, College of Medicine, Jazan University, GGGD6622, Jazan 45142, Saudi Arabia; 2 Department of Psychology, College of Education, Jazan University, GGGD6622, Jazan 45142, Saudi Arabia; 3 Medical Research Centre, Jazan University, GGGD6622, Jazan 45142, Saudi Arabia

**Keywords:** prevalence, maternal postpartum depression, risk factors, Saudi Arabia

## Abstract

Postpartum depression (PPD) is a serious public health problem in many Middle Eastern countries. Mothers with PPD experience various extreme symptoms that affect their daily lives. This study aims to discover how common PPD is in the Jazan region, the most significant risk factors, and how likely depressed women are to seek help. An observational cross-sectional survey targeting a sample of 444 mothers in their first year after delivery using a pre-tested and validated EDPS standard tool to evaluate the prevalence of postpartum depression amongst them has been conducted. The data was collected and then analyzed using SPSS. Descriptive statistics and inferential statistics were used for data analysis. Multivariate logistic regression was used to assess the risk factors associated with PPD. The results indicate an extremely high prevalence of PPD in Jazan (75.7%). The prevalence of mothers having suicidal ideation was 6.3% quite often, 5.0% sometimes, and 7.9% hardly. Regarding the duration of depression symptoms, 34.78% were less than a month, 20.72% were less than six months, and 13.06% were less than one year. The study shows that the development of depression symptoms occurred within less than a week for 30.4% of the women. The most significant association with PPD was a lack of family support, which significantly increased the risk of PPD (OR = 5.9; *p*-value < 0.001). The mothers who had unexpected pregnancies had a risk of PPD (OR = 2.5; *p*-value < 0.001). Current research has revealed a high prevalence of postpartum depression among mothers in the Jazan region and that it is associated with different risk factors that increase the probability of PPD development. Pregnant women need to raise their awareness about PPD and learn how to avoid or deal with it.

## 1. Introduction

Postpartum depression (PPD) involves a cluster of depressive symptoms prevalent among women during the first year after childbirth [1]. It is the most common, serious, and progressive condition characterized by behavioral issues and disorders ranging from mood to mental changes that women experience after giving birth. The clinical symptoms of PPD usually appear within six weeks of childbirth for 84% of the patients [2]. Mothers with PPD experience extreme anxiety, loss of interest, irritability, sorrow, constant fatigue, exhaustion, reduced concentration, sleep deficit, and appetite disturbance, which may make it difficult for them to complete daily care activities for themselves, their baby, and others [3]. Postpartum affective disorders are divided into postpartum blues, non-psychotic postpartum depression, and puerperal psychosis [4,5].

Globally, PPD prevalence varies widely from region to region, and cultural differences might influence risk factors [6,7,8,9]. A recent meta-analysis of 59 studies of postpartum depression showed a prevalence of 13% [6,7]. PPD is less prevalent in traditional cultural settings such as China. For example, a woman tends to rest in bed for the first four weeks after birth, while household chores and childcare are given by the woman’s mother or relatives [10]. The prevalence of PPD in the Arab Region was 18.7% [11]. In Lebanon, the overall prevalence of PPD was 21% [12]. A study in Jordan found a 22% prevalence of PPD [13]. Another study in Bahrain among 237 women after birth found that the prevalence was 37.1% [14]. In 2012, a study conducted in Qatar showed that the prevalence of PPD was 17.6% [15]. A Saudi study in the Dammam region showed a different stage of depression in Saudi women: 17.8% had PPD, 9.8% of mothers had moderate depression, and 8% had severe depression [6].

The etiology of PPD remains unclear; nonetheless, it has risk factors that are biological, psychological, social, and cultural [4]. The most common risk factors related to postpartum depression are a history of psychopathology, psychological disturbance during pregnancy, and poor social support [6]. In addition, other factors that influence the high rate of PPD development in mothers include cesarean section, a strong preference for the baby’s gender, unplanned pregnancy, and medical complications such as anemia due to iron deficiency and gestational diabetes [4]. The risk of PPD increases in women with a history of depression [16]. Also, factors such as unemployment, low socioeconomic status, low income, a low educational level, and a poor marital relationship are the most significant risk factors that can develop psychological problems in mothers [7,8,11,17]. Pregnancy complications, labor, cesarean section, and insufficient breastfeeding were also reported as significant risk factors [9,18,19]. The research highlighted the vital role of seeking behavior regarding PPD [20,21,22,23]. While some mothers in the postpartum period talk to their family and friends to seek help, most do not seek health professional help. The main barrier to that involves a limited understanding of PPD; women often feel ashamed and embarrassed when they suppose they failed to be good mothers [20].

When PPD is ignored, the symptoms typically endure for seven months, although they can sometimes continue into the second year after a woman has given birth to a child [24]. It is essential to screen for and treat PPD due to its effects on mothers, infants, and other family members. While many studies have been conducted in many parts of Saudi Arabia [6,19,25], the prevalence of PPD in the Jazan region is yet to be determined. The present study took place in a society unlike Western societies regarding religious and social values. Bedouins, peasants, and city inhabitants have historically made up most of Saudi Arabia’s population. However, the idea of paternal kinship is infused with this triad. The extended family is a significant social element in Saudi society, and kinship is widely accepted there [26]. So, this study aims to find out how common maternal postpartum depression is in the Jazan region and what depressed women do to obtain help. 

## 2. Materials and Methods

### 2.1. Study Design, Setting, and Population

This study adopted an observational cross-sectional web-based survey design as this way of data collection is more comprehensive and cheaper to carry out, making it easier to enroll large numbers of participants to collect data repeatedly. On several occasions, the data is captured directly in electronic format [27,28]. This study was conducted in the Jazan region in the southwestern region of Saudi Arabia, which has a population of 1.5 million according to a 2010 census. The study population involved mothers living in the Jazan region who were enrolled using social networks. Mothers whose last delivery was more than a year ago, mothers who live outside the Jazan region, and females without children were all excluded.

### 2.2. Sampling Procedure

For a cross-sectional survey, they were estimated using the following equation: n = [(z)^2^ × P(1 − P)]/α^2^.

The parameters used for sample size calculation were a margin of error of not more than 0.05 and a 95% confidence level. We used an anticipated population proportion (P) of 50% because there was no previous estimate for maternal postpartum depression available for the Jazan region. z = 1.96 for a 95% confidence level. The formula then becomes n = [(1.96)^2^ × (0.5)]/(0.05)^2^ = 400. Accounting for a 10% non-response rate, the estimated sample is increased to 444. The snowball sample was used for convenience by distributing copies of the questionnaire to a group known to us, where they were asked to send it out to their contacts.

### 2.3. Data Collection and Study Tool

The main reason for choosing the web-based survey method is the imposed deadline, where survey development, data collection, and data analysis had to be completed within three weeks. We developed a web-based questionnaire with 33 items using Google Forms as an online survey tool, and the questionnaire was divided into four parts to cover all the study objectives. The first part was for the demographic data, including nationality, marital status, age, number of parities, the last delivery, occupation, and income. The second part includes closed-end questions for the major risk factors (7 items), which are the type and complications of delivery, if they had diabetes, if they had anemia (low HB level), if they received support from her family, if they had planned the pregnancy, and if they were expecting a baby of a different gender. The third part was the Edinburgh post-natal depression scale (EDPS) to assess the depression level with a score of 10, or when they experienced PPD (10 items). Items on the scale correspond to various clinical depression symptoms, such as feeling guilt, sleep disturbance, low energy, anhedonia, and suicidal ideation [28]. The fourth part was responsible for measuring the health-seeking behavior of participants and whether they asked for psychological help, followed up on therapy, and complied with treatment visits.

### 2.4. Data Analysis 

The data analysis was performed using the Statistical Package for the Social Sciences (SPSS) version 24, IBM, Armonk, NY, USA. Data analysis involved descriptive statistics as well as inferential statistics. The analysis included simple tabulation, frequencies, and a chi-squared test to test the association between different variables. Multivariate logistic regression was used to assess the risk factors associated with PPD. The dependent variable in this model is PPD (yes/no), and the independent variables are delivery type, severe complications during delivery, gestational diabetes, anemia during pregnancy, family support, unexpected pregnancy, and unexpected gender. A *p*-value less than 0.05 was used to indicate statistical significance.

### 2.5. Ethical Considerations

Ethical approval was obtained from Jazan University’s Ethical Committee with the reference number REC39/8-S031. The researchers ensured that participants’ information was kept private because they did not have to give any personal information and could choose not to. This was stated in the participant’s recruitment statement for the survey. All information was kept confidential and not accessed except for scientific research.

## 3. Results

A total of 444 postpartum mothers completed the questionnaire. Table 1 shows the socio-demographic characteristics of study participants. The data reveal that 98.9% were Saudi mothers, and 96.8% were married. The age distribution revealed that 60.1% were between the ages of 25 and 39. Regarding the number of deliveries, 31.5% had one delivery, 25.9% had two deliveries, and 16.9% had three deliveries.

Table 2 presents the prevalence and pattern of PPD among mothers. Among the 444 mothers, 75.7% had symptoms of depression and EPDS scores higher than 10, and only 24.3% had not shown depressive symptoms. The table further shows that the prevalence of mothers having suicidal ideation was as follows: 6.3% quite often, 5.0% sometimes, 7.9% hardly ever, and 80.9% said never. Regarding the duration of depression symptoms, it was as follows: 34.78% less than a month, 20.72% less than six months, and 13.06% less than one year. The study shows that the development of depression symptoms occurred within less than a week among 30.4% of the women. As for the history of depression, the study found that the number of participants who had the symptoms after previous deliveries were 227 (51.1%).

Table 2 also demonstrates the health-seeking behavior among the study participants. The result of asking for health care (psychology) was only 6.8% of the 444 participants. A high percentage of 70.0% received follow-up therapy, compared to 30.0% who did not. According to compliance with follow-up, visiting was 56.7% committed, and 43.3% did not commit. Private clinics (46.7%) were the most frequently visited health institutions for psychological counseling, followed by PHCCs and general hospitals (26.7% each). 

**Figure 1:**
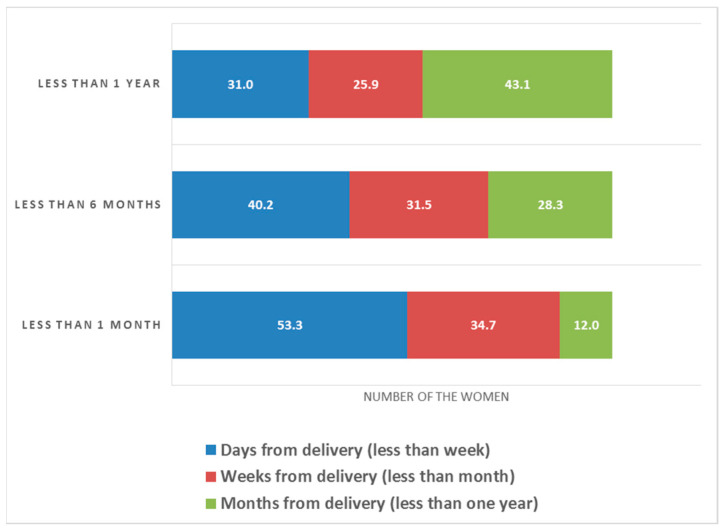
Association between the onset of symptoms and duration of depression symptoms.

According to the findings (Figure 1), 53.33% of the 150 mothers who had symptoms for less than one month had symptoms within days of delivery, 34.67% had symptoms weeks after delivery, and 12% had symptoms months after delivery. Furthermore, another group of mothers (92) experienced symptoms that lasted less than six months; 40.22% of the mothers experienced the symptoms days after the delivery, 31.52% experienced them weeks after delivery, and 28.26% experienced them months after delivery. Nevertheless, another group of 58 mothers experienced less than one year of symptoms. A total of 43.10% of the mothers felt the symptoms months after delivery, 31.03% felt the symptoms days after delivery, and 25.86% felt them weeks after delivery.

Table 3 shows the risk factors associated with PPD among the study participants, as analyzed using logistic regression. Family support has a strong effect on PPD (*p*-value < 0.001), and mothers without family support have a higher risk of developing PPD (OR = 5.9) than mothers with family support. Of the mothers who had unexpected pregnancies, 68.0% had PPD. The result found that unplanned pregnancies had a substantial risk of PPD (OR = 2.5; *p* < 0.001).

## 4. Discussion

This study contributes to investigating the prevalence and risk factors of PPD among women in the Jazan region for the first time. The findings of this study indicate that PPD is extremely common among women in the Jazan region, where 75.7% of the mothers were depressed. This result demonstrates that PPD is one of the most common psychological problems in Jazan. In addition, the prevalence of PPD in Jazan is one of the highest worldwide, as it is higher than that in Morocco (18.7%), Lebanon (26%), Jordan (22%), Bahrain (37.1%), Qatar (17.6%), and Dammam, (Saudi Arabia) (17.8%) [6; 11; 15]. According to the literature, only Kundapur, India, has a higher prevalence (81%) than the Jazan region [8]. Therefore, the high frequency of PPD in our region is due to the fact that most people believe that experiencing symptoms of depression after childbirth is normal and are unaware of this condition and its severe implications, such as suicidal thoughts or injury to the infant. According to earlier research, it is now time to examine distinct cultural customs and beliefs and their effects on PPD rather than only focusing on the prevalence, incidence, and descriptive studies of other cultures [29]. Even though this study was conducted in a religious setting, it was previously believed that higher levels of religiosity would be linked to a decreased incidence of postpartum depression symptoms. This theory was supported by the observation that more traditional religious civilizations have more unified social structures, a stronger ceremonial focus, more distinct roles, and more inclusive community support [30]. Estimates place the prevalence of PPD anywhere from 0.5 to 60.8% across the globe. It is estimated that 19.8% of the population lives with this condition in low- and middle-income countries [31,32].

In terms of how long the depressive symptoms persisted, they did so for an average of 34.78% less than a month, 20.72% less than six months, and 13.06% less than a year. When PPD is neglected, symptoms normally last for seven months, however, they can last into a woman’s second year after giving birth [24].

The present study revealed that only 6.8% of depressed women requested health care from health professionals, which is a meager percentage. Similarly, a study conducted in Riyadh, Saudi Arabia, aimed to examine the impact of the behavior of seeking general health care services and partner support on PPD, and they found a negative relationship between them [24]. In Ethiopia, 6.8% of women who had symptoms of depression requested assistance from a health professional, and 12.7% asked for medical attention from a biomedical care services provider. Only 4.2% of women (*n* = 15) referred to mental health care services [22]. Similarly, in the USA, two out of three Vietnamese-American mothers with PPD reported receiving any mental health care [33]. However, unfortunately, these findings do not provide a clear association between PPD and ever receiving any mental health care due to the small sample size [28]. 

Logistic regression modeling was performed to understand the relationship between risk factors and PPD among the study participants. Family support has a strong effect on PPD (*p* < 0.001), and mothers without family support have a higher risk of developing PPD (OR = 5.9) than mothers with family support. Family support is also significantly associated with PPD prevalence in Morocco [11]. In addition to family support, unexpected pregnancies show the same effect in Qatar [15]. The results found that unplanned pregnancies had a substantial risk of PPD (OR = 2.5; *p* < 0.001). In India, a study showed an association between PPD and the type of infant delivery and employment status, which shows that housewives had a higher liability to show PPD symptoms, and having an infant of an unexpected gender had the same impact. In Iran, type of delivery, low socioeconomic status, and low educational level were the most significant risk factors associated with PPD [17]. The factors that have a negative association with PPD include severe complications during delivery, a low financial status, which, in India [8], was the most reported risk factor, and anemia during pregnancy, even though in Riyadh [19], anemia during pregnancy was a risk factor for PPD; correspondingly, in Qatar [15], anemia had been noticed too, as had gestational diabetes.

The current study has certain limitations. First, with this data collection procedure, we have less control over who receives a copy of the survey; therefore, it is impossible to assess the response rate. Second, it is restricted to Internet users exclusively. Third, because this is a cross-sectional study, the discussed associations and risk variables should be considered cautiously. Fourth, this research only looked at female participants who were able to read. It did not investigate moms who were unable to read, despite the fact that this is a significant population. Because both the exposure and the result are evaluated at the same time in a cross-sectional research design, there is typically no indication of a temporal link between the two variables. This is the fifth and last limitation of the cross-sectional study design. It is not feasible to establish a real cause-and-effect relationship if longitudinal data are not included in the analysis.

## 5. Conclusions

According to the results of our research, the Jazan region has a notably high prevalence of postpartum depression (PPD) among its female population. In addition, we found that this disease was associated with a number of risk factors that increased the probability of developing PPD. This study also revealed that a significant proportion of women do not seek medical care to address PPD, which may result in an even greater load for medical services. The presence of a supportive family and an unplanned pregnancy is considered to be the two most significant risk factors for postpartum depression (PPD). The data presented here can be utilized by health decision-makers, health educators, and midwives to educate moms about postpartum depression (PPD) and better prepare them for the possibility of experiencing it.

## Figures and Tables

**Table 1:** Socio-demographic characteristics of the study sample.

Characteristic	Categories	Frequency	%
Nationality	Saudi	439	98.9
	Non-Saudi	5	1.1
Marital status	Married	430	96.8
	Divorced	8	1.8
	Widow	6	1.4
Age group (years)	24 or less	129	29.1
	25–39	267	60.1
	40–54	48	10.8
Number of deliveries	1	140	31.5
	2	115	25.9
	3	75	16.9
	4	47	10.6
	5	34	7.7
	More than 5	33	7.4
Last delivery	<1 month	38	8.6
	<6 months	117	26.4
	<12 months	289	65.1
Working status	Housewife	292	65.8
	Working outside	152	34.2
Monthly income	less than 5000	178	40.1
	5000–9999	168	37.8
	10,000–14,999	79	17.8
	15,000–19,999	11	2.5
	20,000 or more	8	1.8

**Table 2:** Prevalence, pattern and health care seeking behavior regarding PPD.

Variables	Categories	N	%
Prevalence of PPD using EPDS	Having the symptoms	336	75.7
	Free of the symptoms	108	24.3
Thought of harming self	Never	359	80.9
	Hardly ever	35	7.9
	Sometimes	22	4.95
	Yes, quite often	28	6.3
Duration of PPD symptoms among mothers	less than month	150	34.8
	less than 6 months	92	20.8
	less than 1 year	58	13.1
	None	144	32.4
Onset of symptoms	Days from delivery (less than a week)	135	30.4
	Weeks from delivery (less than a month)	96	21.6
	Months from delivery (less than one year)	69	15.5
Had the symptoms previously after delivery	Yes	227	51.1
	No	217	48.9
	More than 5	12	2.6
Number of deliveries with symptoms	First delivery	131	29.6
	Others	128	28.8
	First delivery + other	185	41.6
Had the symptoms previously after delivery and now depressed	Yes	389	87.7
	No	280	63.1
Ask for health care (psychology)	Yes	30	6.8
	No	414	93.2
Follow-up of therapy	Yes	311	70.0
	No	133	30.0
Compliance with follow-up visiting	Yes	252	56.7
	No	192	43.3
Type of health institutions visited	Primary Health Care Centre (PHC)	119	26.7
	General hospital	119	26.7
	Private clinic	207	46.7

**Table 3:** Logistic regression model for factors associated with the PPD.

Risk Factors	Variable	%	PPD Symptoms (%) EPDS ≥ 10	OR	*p*-Value
Delivery type	Normal	77.25	74.6	1.3	0.347
	Cesarean	22.75	79.2		
Severe complications during delivery	Yes	11	83.7	0.5	0.167
	No	89	74.7		
Gestational diabetes	Yes	7.21	81.3	0.7	0.441
	No	92.79	75.0		
Anemia during pregnancy	Yes	34.68	79.2	0.7	0.191
	No	65.32	73.1		
Family support	Yes	81.98	71.7	5.9	<0.001
	No	18.02	93.8		
Unexpected pregnancy	Yes	52.03	68.0	2.5	<0.001
	No	47.97	84.0		
Un-expected gender	Yes	41.00	72.0	1.4	0.112
	No	59.00	78.2		

## Data Availability

Data are available upon reasonable request.

## References

[1] Zinga D., Phillips S.D., Born L. (2005). Postpartum depression: We know the risks, can it be prevented?. Braz. J. Psychiatry.

[2] O’Hara M.W. (2009). Postpartum depression: What we know. J. Clin. Psychol..

[3] Epperson C.N., Huang M.-Y., Cook K., Gupta D., Chawla A., Greenberg P.E., Eldar-Lissai A. (2020). Healthcare resource utilization and costs associated with postpartum depression among commercially insured households. Curr. Med. Res. Opin..

[4] Stewart D.E., Robertson E., Dennis C.-L., Grace S.L., Wallington T. (2003). Postpartum Depression: Literature Review of Risk Factors and Interventions.

[5] Lobel M., Ibrahim S.M. (2018). Emotions and mental health during pregnancy and postpartum. Women’s Reprod. Health.

[6] Alasoom L.I., Koura M.R. (2014). Predictors of postpartum depression in the eastern province capital of Saudi Arabia. J. Fam. Med. Prim. Care.

[7] Bina R. (2008). The impact of cultural factors upon postpartum depression: A literature review. Health Care Women Int..

[8] Upadhyay R.P., Chowdhury R., Salehi A., Sarkar K., Singh S.K., Sinha B., Pawar A., Rajalakshmi A.K., Kumar A. (2017). Postpartum depression in India: A systematic review and meta-analysis. Bull. World Health Organ..

[9] Mathisen S.E., Glavin K., Lien L., Lagerløv P. (2013). Prevalence and risk factors for postpartum depressive symptoms in Argentina: A cross-sectional study. Int. J. Women’s Health.

[10] Klainin P., Arthur D.G. (2009). Postpartum depression in Asian cultures: A literature review. Int. J. Nurs. Stud..

[11] Agoub M., Moussaoui D., Battas O. (2005). Prevalence of postpartum depression in a Moroccan sample. Arch. Women’s Ment. Health.

[12] Chaaya M., Campbell O., El Kak F., Shaar D., Harb H., Kaddour A. (2002). Postpartum depression: Prevalence and determinants in Lebanon. Arch. Women’s Ment. Health.

[13] Mohammad K., Gamble J., Creedy D. (2011). Prevalence and factors associated with the development of antenatal and postnatal depression among Jordanian women. Midwifery.

[14] Al Dallal F., Grant I. (2012). Postnatal depression among Bahraini women: Prevalence of symptoms and psychosocial risk factors. East. Mediterr. Health J..

[15] Bener A., Burgut F.T., Ghuloum S., Sheikh J. (2012). A study of postpartum depression in a fast developing country: Prevalence and related factors. Int. J. Psychiatry Med..

[16] Pataky E.A., Ehlert U. (2020). Longitudinal assessment of symptoms of postpartum mood disorder in women with and without a history of depression. Arch. Women’s Ment. Health.

[17] Taherifard P., Delpisheh A., Shirali R., Afkhamzadeh A., Veisani Y. (2013). Socioeconomic, psychiatric and materiality determinants and risk of postpartum depression in border city of Ilam, Western Iran. Depress. Res. Treat..

[18] Josefsson A., Angelsiöö L., Berg G., Ekström C.-M., Gunnervik C., Nordin C., Sydsjö G. (2002). Obstetric, somatic, and demographic risk factors for postpartum depressive symptoms. Obstet. Gynecol..

[19] Alharbi A.A., Abdulghani H.M. (2014). Risk factors associated with postpartum depression in the Saudi population. Neuropsychiatr. Dis. Treat..

[20] Corrigan C.P., Kwasky A.N., Groh C.J. (2015). Social support, postpartum depression, and professional assistance: A survey of mothers in the Midwestern United States. J. Perinat. Educ..

[21] Reid K.M., Taylor M.G. (2015). Social support, stress, and maternal postpartum depression: A comparison of supportive relationships. Soc. Sci. Res..

[22] Azale T., Fekadu A., Hanlon C. (2016). Treatment gap and help-seeking for postpartum depression in a rural African setting. BMC Psychiatry.

[23] Sword W., Busser D., Ganann R., McMillan T., Swinton M. (2008). Women’s care-seeking experiences after referral for postpartum depression. Qual. Health Res..

[24] Chrzan-Dętkoś M., Murawska N., Walczak-Kozłowska T. (2022). ‘Next Stop: Mum’: Evaluation of a Postpartum Depression Prevention Strategy in Poland. Int. J. Environ. Res. Public Health.

[25] Ochsenwald W.L., Teitelbaum J. “Saudi Arabia”. Encyclopedia Britannica. https://www.britannica.com/place/Saudi-Arabia.

[26] Almutairi A.F., Salam M., Alanazi S., Alweldawi M., Alsomali N., Alotaibi N. (2017). Impact of help-seeking behavior and partner support on postpartum depression among Saudi women. Neuropsychiatr. Dis. Treat..

[27] Abdollahi F., Etemadinezhad S., Lye M.-S. (2016). Postpartum mental health in relation to sociocultural practices. Taiwan. J. Obstet. Gynecol..

[28] Schleyer T.K., Forrest J.L. (2000). Methods for the design and administration of web-based surveys. J. Am. Med. Inform. Assoc..

[29] Wyatt J.C. (2000). When to use web-based surveys. J. Am. Med. Inform. Assoc..

[30] Dankner R., Goldberg R.P., Fisch R.Z., Crum R.M. (2000). Cultural elements of postpartum depression. A study of 327 Jewish Jerusalem women. J. Reprod. Med..

[31] Halbreich U., Karkun S. (2006). Cross-cultural and social diversity of prevalence of postpartum depression and depressive symptoms. J. Affect. Disord..

[32] Ing H., Fellmeth G., White J., Stein A., Simpson J.A., McGready R. (2017). Validation of the Edinburgh Postnatal Depression Scale (EPDS) on the Thai-Myanmar border. Trop. Doctor.

[33] Ta Park V.M., Goyal D., Nguyen T., Lien H., Rosidi D. (2017). Postpartum traditions, mental health, and help-seeking considerations among Vietnamese American women: A mixed-methods pilot study. J. Behav. Health Serv. Res..

